# Acceptability of an Integrated Model of Mental Health and PrEP Service Delivery for South African Adolescent Girls: Qualitative Results from a Pilot Hybrid Effectiveness-Implementation Randomized Trial

**DOI:** 10.1007/s10461-025-05007-z

**Published:** 2026-02-16

**Authors:** Lisa Mills, Sinead Delany-Moretlwe, Makhosazane Nomhle Ndimande-Khoza, Hlukelo Chauke, Nikita Govender, Nicole Poovan, Ruth Verhey, Dixon Chibanda, Connie Celum, Jennifer Velloza

**Affiliations:** 1 Wits RHI, Faculty of Health Sciences, University of Witwatersrand, 22 Esselen Street, Johannesburg, 2000 Republic of South Africa; 2 Friendship Bench Program, Harare, Zimbabwe; 3 Department of Global Health, University of Washington, Seattle, WA USA; 4 Department of Medicine, University of Washington, Seattle, WA USA; 5 Department of Epidemiology, University of Washington, Seattle, WA USA; 6 Department of Epidemiology & Biostatistics, University of California San Francisco, San Francisco, CA USA

**Keywords:** HIV, Pre-exposure prophylaxis, Mental health, Adolescent girls and young women, Acceptability, Hybrid trial

## Abstract

Adolescent girls and young women (AGYW) at elevated HIV risk often experience common mental disorders (CMD), which are linked to lower PrEP adherence. We piloted a CMD intervention, the Youth Friendship Bench SA (YFB), and delivered alongside PrEP to improve CMD symptoms and PrEP adherence among South African AGYW. From April 2023-February 2024, 116 AGYW (18–25 years) with CMD symptoms were randomized to YFB plus standard of care (SOC) or SOC alone and followed for 12 weeks. In-depth interviews were conducted with 30 AGYW and 13 study staff and key informants. CMD screening alone was seen as beneficial, but referral uptake was low due to perceived severity thresholds, staff attitudes, and logistical barriers. YFB was highly acceptable to AGYW, who valued the intervention for being confidential, informal, and empowering. Integrated CMD and PrEP services were acceptable, and lay counsellor delivery shows promise for addressing the mental health care gap.

## Introduction

Adolescent girls and young women (AGYW, defined as females aged 15–24 years) are a priority population for HIV prevention intervention approaches [1]. Oral pre-exposure prophylaxis (PrEP) has demonstrated high efficacy and has been rapidly scaled up for delivery in South African primary healthcare (PHC) clinics since 2016 [2]. Despite high uptake, AGYW have difficulty adhering to oral PrEP. Studies among Sub-Saharan AGYW show 24–47.2% PrEP adherence at month 3 or 6 [3–5].Other studies have reported overall PrEP adherence at 30–40% for Sub-Saharan AGYW [6, 7]. This may be in part due to mental health challenges. Adolescence is a critical time of onset for common mental disorders (CMD), including depression, anxiety, and post-traumatic stress [8]. Prior HIV prevention studies have shown that 20–50% of AGYW initiating PrEP have moderate to severe CMD symptoms [9, 10]. These symptoms persist in a majority of AGYW despite attending routine healthcare visits for HIV prevention and sexual and reproductive health services, and have been shown to be associated with 25% lower PrEP adherence among AGYW [9].

The intersecting HIV and CMD burden among AGYW and the negative impact of CMD symptoms on PrEP adherence highlight a need for integrated delivery of mental health interventions with PrEP as part of comprehensive primary healthcare (PHC). PHC facilities, where the majority of South Africans access healthcare, are charged with conducting mental health screening, assessment, diagnosis and management, according to South African Adult Primary Care (APC) Guidelines [11]. However, screening is not routinely conducted and most South Africans with a mental health condition do not receive assessment or treatment, in part due to a paucity of trained providers and limited budget for mental health in PHC [12, 13].

Task shifting of delivery of evidence-based psychological interventions from providers to trained non-specialist workers could address this challenge [12, 14]. Cognitive behavioural therapy (CBT) and problem-solving therapy (PST) are psychotherapy approaches recommended by the WHO for mild-to-moderate CMD symptoms that can be delivered by lay counsellors [1, 15, 16]. A 2013 review of 10 trials involving 18,738 women found that psychological and health promotion interventions for perinatal common mental disorders delivered by non-specialist providers in low- and middle-income countries (LMICs) reduced symptom severity compared to usual care (pooled effect size − 0.34), with psychological interventions showing the strongest effects [15]. A later review of 27 trials of mental health counselling interventions delivered to adults by non-specialist providers (predominantly community health workers or peers) in LMICs showed moderate to strong effectiveness improvement in mental health symptoms, with a pooled effect size of 0.49 [16].

The Friendship Bench is a PST intervention specifically developed for lay counsellor delivery in low-resource African settings and significantly improved CMD symptoms among adults in HIV and PHC facilities in Zimbabwe [17]. Lay counsellors can be defined as health workers or volunteers that do not have any formal or specialized professional certification in mental health or counselling [15]. This can include health workers that have professional health care training (e.g., nurses, or doctors), however in this context we refer to lay counsellors as lay or non-professional healthcare workers without formal professional or para-professional certification, nor a relevant tertiary education degree. The Friendship Bench comprises 4–6 individual counselling sessions and an optional economic empowerment group session delivered by lay counsellors. During the first session counsellors guide participants in discussing challenges affecting their mental health, identifying a specific challenge, brainstorming implementable solutions, and developing an action plan for the solution. At subsequent sessions, counsellors check in on participant’s progress in implementing their action plan and guide participants in identifying new challenges.

We previously adapted the Friendship Bench intervention and its delivery approach for AGYW seeking PrEP in formative work with AGYW, healthcare providers, policy makers, and implementers in Johannesburg [18, 19]. We then conducted a randomized pilot trial of our adapted “Youth Friendship Bench SA” (YFB) intervention, integrated with PrEP services, among AGYW in Johannesburg [20]. While there was no effect on PrEP adherence by Week 12, the intervention had a short-term effect on PrEP adherence, with improvements in PrEP adherence by Week 4 in the intervention arm compared with the standard-of-care (SOC) arm. PrEP adherence remained steady in both arms (tenofovir (TVF) detected in urine samples among 47.0% of participants at Week 4 and 44.3% of participants at Week 12). Although there was no intervention effect on CMD symptoms by Week 12, CMD symptoms significantly improved in the cohort over time [20].

In this qualitative sub-study, we explored AGYW and stakeholders’ experiences with the YFB intervention, to contextualize our trial findings and aid in understanding the acceptability, feasibility, and appropriateness of this approach to integrated CMD and PrEP service delivery. More broadly, we also explored implementation gaps in delivery of SOC mental health services in resource-limited settings and perception of task-shifting for mental health and PrEP adherence support.

## Methods

### Study Design

The “Combining HIV prevention Options with Mental health service delivery for Adolescents” (CHOMA) hybrid effectiveness-implementation study aimed to evaluate preliminary effectiveness, acceptability, feasibility, and appropriateness of our YFB intervention to address CMD and PrEP adherence among South African AGYW. CHOMA enrolled 116 AGYW aged 18–25 years old who were living in Johannesburg, able to speak English or isiZulu, using PrEP, and had a score of ≥ 7 on the Self-Reporting Questionnaire-20 item (SRQ-20), which has been validated for detection of CMD symptoms globally [21]. Those with active, unmanaged mental health disorders or suicidal intent were excluded and referred for immediate care.

Participants were randomised 1:1 to either the YFB intervention plus SOC mental health services (screening and referral according to South African APC Guidelines [11]) (*n* = 58) or SOC alone (*n* = 58). Intervention components included delivery of five individual counselling sessions, delivered in-person or remotely over the telephone. In-person sessions were conducted in a private space by young female lay counsellors. Counsellors received 2-week training on delivering the Friendship Bench intervention but had no professional or para-professional health certification or any certification in mental health or counselling. Counsellors had completed secondary schooling, and had some work experience in HIV counselling, community health work and/or peer education. Counsellors completed mock counselling sessions and were supervised by a local research psychologist. Debriefing sessions were conducted to ensure ongoing support. To promote continuity, participants were seen by the same counsellor, unless the counsellor was out of office or unavailable at the time. An optional group session was developed to focus on career and financial planning and participants were offered the option to attend via Zoom or in-person. Intervention participants were offered one-way SMS visit reminders.

All participants were followed up for 12 weeks for three in-person study visits at enrolment, Week 4, and Week 12. Participants receiving the YFB intervention were asked to attend two additional counselling visits, in-person or remotely over telephone, at Weeks 2 and 8. Study staff adhered to the research site’s standard operating procedure for remote visits, including being asked by counsellors to ensure they were in a private space before beginning the session.

Participants received PrEP from their chosen local PHC or were provided with PrEP at the Wits RHI Ward 21 Clinical Research Site following national PrEP guidelines [18]. All participants received brief PrEP adherence counselling at their Week 4 and 12 visits based on a urine point-of-care (POC) assay that detects TFV in the prior 2–7 days. POC results were used to provide motivational adherence counselling: those with detectable TFV were encouraged to continue their PrEP use and those with undetectable TFV were asked whether they wished to continue PrEP and how they could be supported. Co-primary outcomes measured at Week 12 were the proportion of participants with detectable TFV using the urine POC assay, and proportion with reduced symptoms of CMD measured by SRQ-20 scores < 7.

### Qualitative in-Depth Interview Participants

We conducted in-depth interviews (IDI) with a subset of CHOMA study participants (*n* = 30), study staff (*n* = 4), and other key informants (KI) (*n* = 9) involved in mental health and PrEP service delivery. We purposively sampled CHOMA participants by arm (SOC *n* = 14; YFB = 16). Study staff invited participants for interviews at Week 4 and 12 visits, until the target sample was reached (*N* = 30). Interviews were conducted at the visit or scheduled for a time between Weeks 4 and 12. Conducting interviews during study visits assisted in capturing real time experiences, mitigating recall bias, and reducing participant visit burden. KI were approached by phone or email and were purposively identified based on their public, private or non-profit sector PrEP or mental health service delivery experience. Study staff that delivered the YFB were also included. AGYW were reimbursed for time, effort and travel expenses in alignment with South African Health Products Regulatory Authority guidelines. There were no expenses to clinic staff and key informants as interviews were conducted during working hours (clinic staff) or at a self-selected, convenient time and place (key informants). Clinic staff and key informants were offered a small gift of chocolates in appreciation for their participation. All reimbursements and gifts were reviewed and approved by University of the Witwatersrand Human Research Ethics Committee.”

### Data Collection

Experienced female qualitative researchers (HC, NNM) conducted interviews. Structured interview guides for AGYW assessed perceptions and experiences of YFB intervention delivery and urine TVF POC adherence counselling. Experiences and perceptions of SOC services for PrEP and mental health were also explored. Clinic staff interview guides assessed perceptions of the intervention, experiences with delivering the intervention and PrEP motivational counselling, and strategies for future implementation. KI guides assessed perceptions of PrEP delivery, mental health SOC, urine assay-based motivational PrEP adherence counselling, YFB intervention, and strategies for implementing the intervention and integrated care. Interviews were conducted in English, isiZulu, Sesotho, Xitsonga or Setswana, based on participant preferences, and were approximately 60 min long. Interview notes were taken for participants that declined recording (one study staff member and one KI). The remaining interviews were audio-recorded, transcribed, and translated into English. Translations and transcriptions were checked by the study team for accuracy.

### Data Analysis

Experienced qualitative researchers (LM, JV, NMN, HC, NG) analysed transcripts using a qualitative rapid analysis approach [22–24]. Researchers deductively created summary sheet templates for AGYW, KI and staff interviews. The domains on the summary sheets aligned to main topics in each of the interview guides. The team piloted one summary sheet for each category (AGYW, key informant and study staff) to confirm appropriateness and reliability. Transcripts were then assigned to researchers for summary sheet completion. Finally, matrices were developed to organise the data, with participants represented in rows, and domains organised in columns. Summary sheets were reviewed by JV and LM and cross referenced with transcripts to ensure reliability. The team generated analytic memos to extract key themes. Illustrative quotes are referenced with participant identification numbers.

***Ethical Review.***

The protocol was approved by ethics committees at the University of Witwatersrand (Reference number 221006) and the University of California, San Francisco (Reference number 22–37,680). Participants provided written informed consent in English or isiZulu. The clinical trial is registered on ClinicalTrial.gov (NCT05664490).

## Results

We enrolled 30 AGYW in IDI (Table 1). The median age was 21 years (IQR: 19.2–23.0). Most lived with parents or siblings (18/30, 60%) and half (15/30, 50%) were unemployed. The median SRQ-20 score of 8 (IQR: 7.0–10.8) in the qualitative sample was consistent with the score in the overall cohort (9; IQR: 7.0–10.0). We interviewed four study staff members involved in delivering the SOC and YFB interventions and nine KI. KI included nurses, social workers, and psychologists with experience delivering mental health (*n* = 5), PrEP services (*n* = 2) or both mental health and ART/PrEP services (*n* = 2) to youth. The majority delivered services at PHC level, and the remaining KI delivered services at tertiary education institutions (*n* = 2) and privately (*n* = 1). Three KI worked in primary healthcare clinics where study participants were referred to for mental health services. Interviews were conducted from June 2023 to March 2024.

**Table 1:** Enrollment demographic, behavioral, and mental health characteristics of qualitative participants and overall sample

	N (%) or median (IQR)^1^	
	Qualitative Sample *N* = *30*	Total Sample *N* = *115*
*Study Characteristics*		
Study arm Intervention^2^ Standard of Care	16 (53.3%) 14 (46.7%)	57 (49.6%) 58 (50.4%)
*Demographics*		
Age (median), years (IQR)	21.0 (19.2–23.0)	21.0 (20.0–23.0)
Has romantic or sexual partner	26 (86.7%)	95 (82.6%)
Education level No secondary schooling Secondary school completed At least some post-secondary schooling	1 (3.3%) 19 (63.3%) 10 (33.3%)	1 (0.9%) 74 (64.3%) 40 (34.8%)
Unemployed	15 (50.0%)	54 (47.0%)
Living with parents or siblings	18 (60.0%)	79 (68.7%)
*Mental Health*		
SRQ-20^3^ score	8.0 (7.0–10.8)	9.0 (7.0–10.0)
PHQ-9^4^ score	9.0 (7.0–10.8)	8.0 (5.5–11.5)
Anxiety symptoms^5^	7.0 (6.0–10.8)	6.0 (4.5–10.0)
Positive post-traumatic stress screen^6^	15(50.0%)	48 (41.7%)
Positive alcohol or drug use screen^7^	8 (26.7%)	42 (36.5%)
Ever experienced physical, sexual, or emotional gender based violence^8^	21 (70.0%)	85 (73.9%)
Coping self-efficacy score^9^	19.0 (16.0–23.5)	18.0 (15.0–23.0)
Self-esteem score^10^	28.5 (24.5–33.0)	27.0 (24.0–30.5)
*Sexual Behavior*		
Condom use during vaginal sex in past month^11^	25 (86.2%)	91 (81.2%)
Reported transactional sex in the last month^12^	7 (23.3%)	22 (19.1%)
*PrEP Behavior*		
Initiated PrEP ≥ 3 months prior to enrollment	3 (10.0%)	6 (5.2%)

^1^Data are presented as median (interquartile range) for continuous variables and frequency (percentage) for categorical variables

^2^58 randomized to intervention but only 57 completed demographic information

^3^Possible range 0–20. A higher score indicates more depressive symptoms

^4^Possible range 0–27. A higher score indicates more depressive symptoms

^5^Anxiety symptoms assessed by GAD-7. Possible range 0–21. A higher score indicates more anxiety symptoms

^6^Post-traumatic stress assessed by PC-PTSD 4 item checklist. Positive screen if at least 3 items are endorsed

^7^Alcohol and drug use assessed by CAGE (4 items). Positive screen if at least 2 items are endorsed

^8^Questions based on WHO screening tool

^9^Assessed by Coping Self Efficacy (CSE) Scale. Possible range 0–39. A higher score indicates better coping self-efficacy

^10^Assessed by Rosenburg Self-Esteem Scale. Possible Range 10–40. A higher score indicates higher self-esteem

^11^1 missing value for qualitative participants; 3 missing values for full sample

^12^Includes sex in exchange for food, clothes/accessories/cosmetics, cell phone, items for children or family, transport, own school or residence fees, somewhere to stay, or cash

### Perceptions of Youth Friendship Bench SA Problem-Solving Sessions

All YFB participants (*n* = 16) felt that the intervention was beneficial, reporting that the counselling had helped them manage stress and anger, feel more positive or ‘at peace’, and improved their confidence, self-understanding and self-esteem. Participants explained that they became more open to talking about challenges and seeking care for mental health (There is no small or big problem; if you have a problem, you should talk so you can have a way forward on which steps you have to take.” (240,081, YFB, SRQ-20 at enrolment = 8). A few participants mentioned that the first session was particularly helpful as they were able to talk about challenges and express their emotions (“That’s when I poured my whole heart out,” 240,006, YFB, SRQ-20 at enrolment = 7).

Improved problem-solving, self-reliance and decision-making were identified as central parts of intervention acceptability and success. Participants detailed that the sessions taught them to break down problems into smaller pieces, come up with their own solutions, plan ahead and address challenges step-by-step (“This Friendship Bench made me to realize that I myself have answers,” 240,094, YFB, SRQ-20 at enrolment = 13). Many participants shared that they were able to successfully implement solutions to their identified challenge.

Participants valued the confidentiality and informality of counselling sessions, and some expressed that their initial anxiety about privacy or sharing intimate details was allayed by the counsellor’s communication skills, non-judgment and a friendly and informal atmosphere.

“It has been great because it’s not too formal, it’s like talking to a friend, but it’s much better because it’s a safe space and its great talking to someone who is not your friend, someone who can be neutral when giving you advice because they don’t know you.” (240068, YFB, SRQ-20 at enrolment=11)

KI and study staff were positive about the patient-orientated problem-solving approach, and felt that it was beneficial, empowering and offered a good framework for follow up of patients. Study staff felt that the YFB provided focus during sessions, and this avoided participant fatigue from ‘going in different directions.’ The value of providing a safe space to young women was also highlighted.

“We create safe spaces for them because we know that it’s not easy for them to talk about their challenges with their friends, they have been telling us that whenever they talk about their issues with their friends, they [friends] go out and tell other people. With us, we create a safe environment for them, we tell them that everything is strictly confidential, so they share everything without any concerns.” (S002, study staff delivering YFB)

Study staff expressed that some participants were not able to implement their plan, and sometimes relied on external factors which could cause delays (e.g., a family member’s availability or financial assistance). One study staff member suggested that coordinating counselling sessions with PrEP refills might allow more time for participants to implement their plans (routine PrEP refills are at week 4 and every 12 weeks thereafter, while YFB sessions were more frequent at week 2, week 4, week 8 and week 12).

In anticipation of retention challenges, the adapted YFB intervention incorporated an option for remote counselling sessions at Weeks 2 and 8. Most intervention participants opted for remote counselling, and reported that it was acceptable, created convenience, avoided travel and related expenses, and was more private or comfortable at home.

“I felt like they were just checking up on me to see if I’m coping and if I achieved what I planned. The telephonic counselling gave me more strength to work on my goals. I was happy that there is someone who is checking up on me.” (240041, YFB, SRQ-20 at enrolment=9).

Despite this, most participants said they had experienced challenges during remote counselling, including distraction, noise or lack of privacy at home, feeling worried they would miss the call, feeling they could not open up, and poor connectivity and audibility. Study staff added that remote sessions were generally much shorter than in-person, they were unable to see non-verbal communication, and that participants often seemed rushed, preoccupied, unavailable at the agreed time, and difficult to reach during working hours. KI felt that remote counselling could be acceptable and beneficial for some AGYW, especially where patients have challenges with transport. Access to phones and airtime in public facilities was identified as a potential barrier to implementation.

SMS visit reminders were also included in the adapted YFB intervention in response to anticipated retention challenges. No participants refused the optional SMS visit reminders, and participants shared that they were acceptable and useful reminders that helped them to prepare, plan, and reschedule if needed:

“I’m always busy, I drink a lot as well [laughs] so sometimes, I forget that I have to come to the study, so the time I receive the SMS, I know I have to prepare myself to come to the study.” (240089, YFB, SRQ-20 at enrolment=11)

KI felt that SMS reminders would be useful as patients often lose their appointment cards and forget their clinic dates. Study staff reported that implementation was simple and involved entering the patient’s next visit date into the messaging platform at the end of their visit. Some KI expressed implementation concerns including extra workload, training and access to resources needed to implement this service.

### Youth Friendship Bench SA Group Sessions

Group session attendance was low, with 12 out of 57 participants in the intervention arm attending one of the three group sessions conducted. Of the 16 YFB participants that were interviewed, five had attended group sessions. Participants that attended a group session felt that interacting was enjoyable, and it was helpful hearing about other’s experiences and learning from their ideas.

“Us young women are getting an opportunity to show one another the good things that we have to do and motivate each other.” (240064, YFB, SRQ-20 at enrolment=7).

Study staff and KI agreed that group sessions focused on career and financial planning were appropriate, as many young women face similar struggles with unemployment and many that attended the group session were unemployed. One KI noted that she had referred many young women for job placement, but offering group sessions at the clinic could provide alternative opportunities to invite organisations to the clinic.

“I normally refer them to outreach foundations just here. So, they do have computers and all of that but if we were to get these adolescents and make a group, we can actually invite the people from there to come and present.” (KI002, social worker at PHC).

Study staff reported that participants opted for remote attendance to the group sessions due to lack of transport money but that there were challenges with poor connectivity and work and school commitments. One KI echoed concerns about coordinating groups due to previous experience with Youth Care Clubs for adolescents living with HIV (PLWH), that had been difficult to ‘fill up’ due to young people’s busy schedules. Study staff and young women suggested that facilitating larger or in-person groups may improve engagement in the sessions.

### SOC Mental Health Screening and Referrals

Almost all the AGYW that were interviewed said that they had not received any screening or mental health services prior to joining the study (“No one has ever asked me about how I am feeling,” 240,086, YFB, enrolment SRQ-20 = 14). One participant said that she was not asked about or offered mental health services, even when reporting an incident of domestic violence at her PHC. AGYW did not feel comfortable advocating for mental health services, “Social workers don’t take you seriously if you don’t have a referral,” (240,128, SOC, SRQ-20 at enrolment = 7).

Some participants found CMD screening and referral (SOC) at each study visit beneficial in and of itself, and felt it helped them realize the extent of their mental health challenges, focus on themselves, reach out for support, and feel ‘better’ or more open to seeking mental health services in future:

“I’ve learned that I’m now much open than before. I talk whenever I have something that is bothering me, and I don’t just get angry over small stuff anymore. I just learned to talk about it and if there is a problem, I mostly talk to my dad about it since he’s the only one who takes care of us, so he’s been there for me.” (240012, SOC, SRQ-20 at enrolment=8).

Despite appreciation for CMD screening and referral, some participants declined referral to specialist mental health services, and most that had accepted referral had not actually accessed further services at the time of their interview. A few explained that they could manage independently, felt better after intervention counselling or wanted to first complete the YFB intervention. Others said they had not attended due to time constraints, or concern about travel costs, long clinic visits or waitlists. Many expressed fears of talking about their issues, feeling ‘dumb’, vulnerable, weak, or ‘lectured’ when presenting to a clinic for mental healthcare. For the four participants (two SOC participants and two YFB participants) who did access a social worker or psychologist at their local clinic following referral, they felt that accessing further care was beneficial.

“It was helpful because I got some solutions about how to tackle a situation and how to reach out when something happens.” (240065, YFB, SRQ-20 at enrolment=10)

KI involved in providing mental health services in public facilities acknowledged that mental health screening was not routinely carried out due to capacity and workload, and that mental health referral is at the discretion of nurses. KI at facilities where CHOMA participants were referred stated that the intervention was useful in identifying patients that could benefit from psychosocial or PrEP use support. They also noted the benefit of offering immediate mental healthcare with a lay counsellor:

“When a young person needs attention quite quickly, it’s difficult to get them seen. So, that’s perhaps one of the bottlenecks. In terms of the Friendship Bench specifically, that approach is a good one. Because clearly, we need to be handling more at the level of the clinic, rather than relying on these kinds of upward referrals.” (KI008, clinical psychologist at PHC).

For future implementation, study staff and KI suggested that there be a dedicated PHC staff member to conduct mental health screening, and that those individuals be trained in interpretation of CMD screening assessments and identification of individuals that would benefit from the intervention. KI also expressed that counsellors would need to be familiar and comfortable identifying cases that require escalated care. Established referral pathways and awareness of community resources for both mental health and social services was identified as a necessary component of intervention success, but one that could pose challenges (“In terms of resources, we’re not always aware of where we can refer,” KI008, clinical psychologist at PHC). KI made suggestions to combat potential stigma around CMD screening, including use of posters or video advertisements in waiting areas, a comprehensive introduction to mental health and the benefits of counselling, opportunities for question and answers in a private space, and emphasis on voluntary participation Table 2.

**Table 2:** Illustrative quotes by theme

Theme	Illustrative quote
Youth Friendship Bench SA was highly acceptable, and participants valued confidentiality, informality, and opportunity to talk about mental health	“My information is kept confidential and she’s friendly, there is no small or big problem; if you have a problem, you should talk so you can have a way forward on which steps you have to take.” (240,081, YFB, SRQ-20 at enrolment = 8) “I wasn’t sure of how she was going to react when I talk to her, especially when it involves my sexual life, but besides anything, I know that she’s not going to do anything that is not on the consent form I signed.” (240,034, YFB, SRQ-20 at enrolment = 9)
Problem-solving core components of the Youth Friendship Bench SA helped empower AGYW to solve challenges in their lives	“So, when I first came, I had so much anger, I had stress, so the counsellor helped me with what to do to manage the anger and stress I had. We came up with some plans and solutions that helped me” (240,006, YFB, SRQ-20 at enrolment = 7) “In every session, she [counsellor] asks me if I did the things that I said I was going to do, and she makes me trust on myself in terms of decision making and planning because I’m the one who made all those decisions.” (240,054, YFB, SRQ-20 at enrolment = 10)
Most AGYW had not previously received CMD screening; screening and referral was seen as beneficial	“The favorite part [about SOC mental health service] was talking to someone who can actually listen to you, it’s that thing that you don’t get disturbed, ignored and stuff, so her listening to me was my favorite part” (240,024, YFB, SRQ-20 at enrolment = 9) “I think it’s helpful [referral letter] because it’s hard to get access to psychologists, so having a letter referring you there could make it much more simple”. (240,190, SOC SRQ-20 at enrolment = 8) “The psychologist helped me so much; she talked to me and gave me time to talk about what was inside me.” (240,069, SOC, SRQ-20 at enrolment = 18)
Integrated CMD and PrEP services are needed among this population	“Most women are going through difficult times, mostly women in informal settlements, so I think they are going to accept this approach.” (240,054, YFB, SRQ-20 at enrolment = 10) “Giving young women the tools and skills that they need to be able to deal with problems that they’re facing I think is critical and is one part of the solution. I think the other thing that the intervention answers is, just simply being a space where young women can be heard, be listened to and feel that it’s okay to talk about mental health”. (S004, study staff delivering YFB)
Successful implementation will require deployment of additional staff to implement the intervention and adequate staff training	“I think it will be useful for staff to get experience in that training of being listened to; of both in terms of their concerns about it, and their questions. But also, if it’s possible, like in a role play to give everybody an experience of how powerful it can be to get that kind of problem-solving counselling, so that they can experience for themselves how good it can feel, how beneficial it can feel.” (KI003, counselling psychologist at tertiary institution) “If there will specifically be people who are meant to be counsellors, available to such patients, I think it will also help them a lot. I don’t think there will be any negatives, because it will only benefit us at mental health. It will reduce the work pressure.” (KI005, nurse at PHC)

### Integrated Mental Health and PrEP Services

Participants were supportive of implementing integrated mental health and PrEP services and felt that it was needed for peers that face similar challenges (“I won’t be given [PrEP] pills and go home…I get to talk about what is happening in my life,” 240,081, YFB, SRQ-20 at enrolment = 8). While the results of the trial [17] indicated that there was no intervention effect on PrEP adherence, several participants said that the YFB had motivated them to adhere to PrEP. PrEP adherence issues were not identified as the primary problems that participants chose to work through in their YFB problem-solving sessions. Instead, issues that participants chose to address in the sessions were related to intimate partners, difficult living arrangements, family or social issues, mental health struggles, school applications and employment challenges. A few described the influence of the YFB on their PrEP adherence as indirectly motivating, “I won’t say the counselling sessions affected the way I was taking PrEP, they also encouraged me to continue taking my PrEP.” (240,006, YFB, SRQ-20 at enrolment = 7).”

Study staff and KI felt that integration was acceptable and could address the lack of mental health service delivery for PrEP users but also more broadly for young people accessing primary healthcare. KI highlighted the importance of addressing mental health challenges that impact health-seeking behaviour.

“PrEP is a biological intervention, but the reasons people take it…are mostly psychosocial. It seems impossible to ignore mental health aspects because if someone’s mental health is poor…it’s going to have a huge impact on how they take care of themselves.” (KI003, counselling psychologist at tertiary institution).

Delivery of mental health services by a lay counsellor was reported as an attractive answer to high workloads in public clinics. Many KI emphasized that acceptability of the intervention would depend on additional staff to implement these services, as well as staff ‘buy-in’, additional clinic space, and managing the length of patient visits. They suggested that time commitments are clearly communicated to patients, all clinic staff be trained about the importance of mental health, processes be efficient, and staff roles clearly defined.

Counsellors felt that the training they received was appropriate, and conducting mock counselling sessions had been particularly useful.

“The training prepared me in a way that I learned everything that I’m doing because we had mock sessions where we would be corrected if we forgot something.” (S002, study staff delivering YFB).

One counsellor suggested that more training on dealing with cases of violence or abuse may have assisted as some participants facing these issues had refused upward referral. Counsellors found the regular debriefing sessions to be helpful.

“We have group sessions for us. Debriefing sessions. They are quite helpful because I can see I’m not the only one who is having these challenges. And we try to address them all, our support system.” (S001, study staff delivering YFB).

Study staff reflected that supportive supervision of lay counsellors provided in this study was essential to ensuring that counsellors were facilitating SMART (specific, measurable, achievable, realistic and time-bound) action plans. KI also emphasized the importance of in-service training and added that all clinic staff should be included in training or offered opportunities to address any implementation concerns. It was noted that supervision or training may pose a challenge at facilities where social workers or psychologists attend only weekly, or where staff are regularly rotated.

## Discussion

The YFB intervention was highly acceptable and addressed unmet mental health needs among AGYW PrEP users. The core problem-solving component was empowering for young women, and the confidentiality and friendly atmosphere offered by young female counsellors was highly valued by intervention participants. For stakeholders, delivery of mental health services by dedicated lay counsellors was an attractive answer to high workload burden in PHC facilities, and underserviced mental health needs.

The provision and perceived benefit of SOC CMD screening and upward referral may have influenced the trial outcomes, which showed significant improvement of CMD symptoms in both study arms. The perceived benefit of SOC screening and referral and the improvement in all participant’s CMD symptoms suggest that SOC mental health services, delivered in a youth-friendly manner, may be enough to improve mental health outcomes for some AGYW. Gaps in screening and referral at PHC facilities were identified by AGYW however, and stakeholders attributed these to time constraints and understaffing. The South African health sector’s funding crisis and continued under-investment in operational and staffing needs underscores the need to consider alternative, cost-effective solutions to improving CMD screening in PHC facilities including digital self-screening and administration by non-specialized providers [25].

Our research responds to the call for integrated mental health interventions delivered alongside HIV prevention to improve HIV prevention outcomes. The trial findings showed no impact of the YFB on PrEP adherence [20], and qualitative findings indicated that the beneficial or motivational impact reported by participants was not perceived as direct. Mental health issues may have been prioritized over PrEP adherence—participants selected personal and mental health challenges in their problem-solving sessions rather than PrEP adherence challenges, and participants may have been attracted to the study by the mental health services offered as part of the trial (the majority of both IDI and cohort participants had never initiated PrEP prior to study enrolment).

While the problems discussed (e.g., family stresses, economic challenges) may have had an indirect influence on PrEP adherence, the impact of problem-solving approaches on PrEP adherence may be more detectable after longer periods of time [26]. Some participants reported insufficient time to implement their action plans, and remote counselling sessions were perceived as more convenient, but generally as less engaging. Measuring PrEP adherence after a longer follow up period with in-person counselling sessions that coincide with monthly PrEP refills may be useful for future research assessing the impact of CBT and PST approaches on PrEP adherence.

Study staff and KI identified factors that would enhance feasibility of integrated mental health and PrEP services including deployment of dedicated lay counsellors, establishment of referral pathways, and comprehensive training with ongoing supportive supervision. Feasibility may be enhanced by continued stakeholder and community engagement, and monitoring and evaluation for iterative development and improvement [27].

CBT, PST and task-shifting approaches have been able to provide scalable and effective strategies for addressing mental health challenges and improving medication adherence for other populations including PLWH, with success depending on tailored implementation, cultural sensitivity, manageable case load, and supportive systems for non-specialist health workers [15, 27, 29, 30]. In implementation research among African AGYW living with HIV, patients and health providers indicate that CBT and PST approaches are highly acceptable, feasible, and appropriate for integrated HIV treatment and mental health service delivery [26, 31–35]. Research on the impact of integrated services among African AGYW PrEP users is limited [28]. More evidence is needed to demonstrate the long-term impact of CBT, PST and task-shifted approaches for AGYW PrEP users in LMICs, and to explore interactions and factors that influence efficacy across contexts.

This study had a number of strengths and limitations. Our qualitative sub-sample included representation from both study arms, allowing for comparison of a range of experiences and perspectives. The study staff sample was small relative to the KI sample, however, inclusion of both groups aimed to allow for balanced insights on implementation feasibility. To reduce potential social desirability bias, interviews were conducted by qualitative data collectors who were not part of the intervention delivery team. Several participants were interviewed at their Week 4 visit, helping to reduce recall bias and making it possible to interview participants that did not return for subsequent follow up because of less favorable experiences. The analysis team who developed data summaries included researchers with mixed experience and backgrounds, allowing for cross-verification of themes and interpretations. The study recruited participants from a limited number of partnering PHC facilities, and interviews were conducted in the context of a research study with a short follow up period. Results may therefore not be generalizable to integrated service delivery in more programmatic settings.

## Conclusion

The YFB intervention addressed unmet mental health needs through an empowering, youth-friendly approach delivered by lay counsellors. The intervention was highly acceptable to participants and stakeholders, offering a potentially scalable solution to high workload burdens in PHC facilities. Identified gaps in mental health screening and referral underscore the need for cost-effective strategies including task shifting for mental health services and digital self-screening tools. While the research supports the feasibility and benefit of CBT, PST, and task-shifting for PrEP adherence, further research is needed about the long-term impact on PrEP adherence among AGYW in LMICs, and context-specific factors that may influence outcomes.

## Data Availability

All relevant data are within the manuscript. All deidentified transcripts of interviews are stored at Wits RHI, South Africa. All relevant data are within the manuscript. Data is available to from the study corresponding author on reasonable request. MS Word was used to create summary sheets and MS Excel was used to create the analysis matrix.
